# The stem cell adjuvant with Exendin-4 repairs the heart after myocardial infarction *via* STAT3 activation

**DOI:** 10.1111/jcmm.12272

**Published:** 2014-04-30

**Authors:** Jianfeng Liu, Haibin Wang, Yan Wang, Yujing Yin, Zhiyan Du, Zhiqiang Liu, Junjie Yang, Shunying Hu, Changyong Wang, Yundai Chen

**Affiliations:** a Department of Cardiology, Medical School of Chinese PLA, Chinese PLA General HospitalBeijing, China; b Department of Advanced Interdisciplinary Studies, Institute of Basic Medical Sciences, Tissue Engineering Research Center, Academy of Military Medical SciencesBeijing, China; c Institute of Transfusion Medicine, Academy of Military Medical SciencesBeijing, China

**Keywords:** Exendin-4, adipose-derived stem cell, myocardial infarction, STAT3, paracrine

## Abstract

The poor survival of cells in ischaemic myocardium is a major obstacle for stem cell therapy. Exendin-4 holds the potential of cardioprotective effect based on its pleiotropic activity. This study investigated whether Exendin-4 in conjunction with adipose-derived stem cells (ADSCs) could improve the stem cell survival and contribute to myocardial repairs after infarction. Myocardial infarction (MI) was induced by the left anterior descending artery ligation in adult male Sprague-Dawley rats. ADSCs carrying double-fusion reporter gene [firefly luciferase and monomeric red fluorescent protein (fluc-mRFP)] were quickly injected into border zone of MI in rats treated with or without Exendin-4. Exendin-4 enhanced the survival of transplanted ADSCs, as demonstrated by the longitudinal *in vivo* bioluminescence imaging. Moreover, ADSCs adjuvant with Exendin-4 decreased oxidative stress, apoptosis and fibrosis. They also improved myocardial viability and cardiac function and increased the differentiation rates of ADSCs into cardiomyocytes and vascular smooth muscle cells *in vivo*. Then, ADSCs were exposed to hydrogen peroxide/serum deprivation (H_2_O_2_/SD) to mimic the ischaemic environment *in vitro*. Results showed that Exendin-4 decreased the apoptosis and enhanced the paracrine effect of ADSCs. In addition, Exendin-4 activated signal transducers and activators of transcription 3 (STAT3) through the phosphorylation of Akt and ERK1/2. Furthermore, Exendin-4 increased the anti-apoptotic protein Bcl-2, but decreased the pro-apoptotic protein Bax of ADSCs. In conclusion, Exendin-4 could improve the survival and therapeutic efficacy of transplanted ADSCs through STAT3 activation *via* the phosphorylation of Akt and ERK1/2. This study suggests the potential application of Exendin-4 for stem cell–based heart regeneration.

## Introduction

Acute myocardial infarction (AMI), which is accompanied by irreversible loss of functional cardiomyocytes, adverse cardiac remodelling response and progressive deteriorative heart function, is still a leading cause of morbidity and mortality worldwide [1]. Although clinical methods are available for MI, none of them was so satisfactory. Because of the limited regenerative capacity of adult heart [2], stem cell therapy has been emerging as a novel and prospective therapy for myocardial repairs.

In the past decades, various stem cells have been tried for myocardial repairs, including skeletal myoblasts, embryonic stem cells, mesenchymal stem cells *etc*. [3]. Both animal experiments and clinical studies have demonstrated positive effects in improving heart healing after MI [4,5]. Among the myriad of cell types used for regenerative therapies, adipose tissue–derived stem cells (ADSCs) have recently been considered a promising candidate for myocardial repairs because of the abundance of autologous sources, ease to harvest, cardiomyogenic potential and definite therapeutic effects [6,7]. However, the poor cell viability after transplantation as a result of the hostile engraftment environment largely attenuated the therapeutic effect (*i.e*. ischaemic microenvironment, including oxidative stress, inflammation, low vascularization [3]).

The efficacy of cell therapies largely depends on the appropriate control of the fate and function of the engrafted cells. Therefore, strategies for enhancing stem cell survival, proliferation and differentiation have become one of the hot topics of central interest. The application of adjuvant drug is a promising way to support the stem cell therapy [8]. Adjuvant therapy, such as atorvastatin, simvastatin and hyperbaric oxygen, may play a role in improving harsh microenvironment of ischaemic myocardium, increasing the survival of transplanted stem cells, inhibiting host cell apoptosis and facilitating cardiomyogenesis [9–12].

Exendin-4, isolated from the saliva glands of the Gila monster, has exhibited potent anti-diabetic activities through glucagon-like peptide-1 (GLP-1) receptor (GLP-1R), which has been detected in heart [13]. It mimics the function of GLP-1 and exerts cardioprotective effects beyond glucose control *via* GLP-1R-dependent and -independent pathways [13,14]. Indeed, exendin-4 possessed pleiotropic effects in cardiac protection after MI, such as potential anti-inflammatory [15], antioxidant [16,17], anti-apoptotic [18], protection of endothelium actions [19]. In particular, GLP-1 is shown to be able to promote the proliferation of mesenchymal stem cells and reduce their apoptosis significantly [20]. These studies suggest that Exendin-4 may benefit stem cell therapy in MI. Nevertheless, no such study has been reported so far to our knowledge.

In this study, we investigated the possible feasibility and efficiency of exendin-4 as an adjuvant drug for ADSC transplantation for MI. Efforts were made to test such hypothesis in the rat models of MI.

## Materials and methods

### Isolation, culture, characterization and lentiviral labelling of rat ADSCs

Adipose-derived stem cells were acquired from inguinal subcutaneous adipose tissue of Sprague–Dawley rat as previously described [21,22]. The immunophenotype of ADSCs, including CD34, CD31, CD45, CD90 and CD29, was analysed by flow cytometer (BD). The verification of osteogenic and adipogenic differentiation was performed by Alizarin Red staining and Oil Red O staining respectively. The ADSCs were lentivirally transduced to express both fluc and mRFP as described previously [21,23]. The 5% highest mRFP-expressing cells were selected by FACScan (BD FACSVantage Diva) and expanded before usage. Details are described in supplementary materials.

### Myocardial infarction model, cell delivery and Exendin-4 treatment

Male Sprague–Dawley (SD) rats (250 ± 10 g) were purchased from the Experimental Animal Center, Academy of Military Medical Science. All procedures were in accordance with the *Guide for the Care and Use of Laboratory Animals* published by the US National Institutes of Health (NIH Publication, 8th Edition, 2011) and approved by the Institutional Animal Care and Use Committee of the General Hospital of Chinese People's Liberation Amy. Rats were randomly divided into the following groups with *n* = 30 each: (*i*) PBS, (*ii*) Exendin-4 only, (*iii*) ADSCs transplantation, (*iv*) ADSCs transplantation combined with Exendin-4.

Rats were intraperitoneally anaesthetized with sodium pentobarbital (30 mg/kg). The animals were then incubated and ventilated by a volume-regulated respirator during surgery. After a left lateral thoracotomy and pericardectomy, the left coronary artery was identified and gently ligated with a 6.0 prolene suture. Successful AMI was confirmed by the typical ST segment elevation in electrocardiography. Immediately after MI, rats were given an intramyocardial injection of labelled ADSCs (100 μl PBS of 5 × 10^6^ ADSCs for ADSCs group or Exendin-4+ ADSCs group) or PBS alone (100 μl for control group) into three injected foci along peri-infarct zone. Injections were verified by a slight lightening in the colour of the myocardium as the solutions entered the infarcted wall. Finally, the chest was closed (detailed in the supplementary materials). During the surgical procedures, the adequacy of anaesthesia was monitored by using absence of the pedal withdrawal reflex, slow constant breathing and no response to surgical manipulation. Buprenorphine was administered before and after the procedures (0.05 mg/kg, i.p). Freshly prepared Exendin-4 (Sigma-Aldrich, St. Louis, MO, USA) was intraperitoneally administered daily at a low dose of 1 nmol/kg starting 3 days prior to MI in the morning for 7 days [12,24]. All rats were treated with cyclosporin A (10 mg/kg/day, ip, Sigma-Aldrich) 2 days before transplantation and daily until the end of the study.

### *In vivo* optical bioluminescence imaging

The correlation between fluc/mRFP- positive ADSCs and *ex vivo* bioluminescence intensity was confirmed by preparing various numbers of cells (per well) in a 96-well plate. Cardiac bioluminescence imaging was performed on all rats receiving ADSCs by using the Xenogen optical macroscopic imaging system by a blinded researcher. Rats were anaesthetized initially with 3.5% isofluorane and maintained with 1.5–2% isofluorane. The adequacy of anaesthesia was monitored by toe pinch. After intraperitoneal injection of the reporter probe D-luciferin (375 mg/kg bodyweight), rats were imaged for 30 min. with 2-minute acquisition intervals until peak signal was observed. For analgesia, buprenorphine (0.05 mg/kg) was given intraperitoneally. The same rats were longitudinally subjected to BLI on days 1, 3, 7, 14, 28 after AMI.

### Echocardiography

Ejection fraction (EF) and fractional shortening (FS) were evaluated in anaesthetized rats (*n* = 12 per group) by echocardiography at 28 days by using a 14-MHz probe as previously described [25]. LV parameters were obtained from M-mode interrogation in a parasternal long-axis view (detailed in the supplementary materials).

### Cardiac viability assessment with micro-PET imaging

Detailed procedures for small animal PET imaging have been reported previously [26,27]. Briefly, Small animal micro-PET imaging (GE Healthcare, Pollards Wood, UK) was performed in a subset of the animals (*n* = 5 per group, chosen randomly) at 4 weeks. After being fasted for 6 hrs, rats were anaesthetized initially with 3.5% isofluorane and maintained with 1.5–2% isofluorane. The adequacy of anaesthesia was monitored by toe pinch. Each rat was injected i.p. with glucose solution to standardize glucose and insulin levels. [^18^F]-fluorodeoxyglucose (FDG) was injected *via* tail vein. Then 10 min. PET scans were performed at 40 min. after injection. For analgesia, buprenorphine (0.05 mg/kg) was given intraperitoneally. Images were performed and analysed by an experienced researcher who was blinded to the groups. For each micro-PET scan, regions of interest (ROIs) were drawn on over survival myocardial coronal images by using image software. Assuming a tissue density of 1 g/ml, the ROIs were converted to MBq/q per min by using a conversion factor and then divided by the administered activity to obtain an imaging ROI derived per cent injected dose per gram (% ID/g).

### Sample preparation and histological analysis

The animals were killed after functional measurements with an overdose of sodium pentobarbital in compliance with above guidelines. Their hearts were excised and rapidly frozen in O.C.T medium for the preparation of frozen sections (4 μm thickness). Ten sections were prepared at 10 different transversal levels at the site of tissue necrosis, equally distributed from base to apex. At 1 week after transplantation (*n* = 6/Groups, chosen randomly), dihydroethidium (DHE) staining and TUNEL staining were performed to assess the intracellular reactive oxygen species (ROS) level and apoptosis. At the end of the experiments (*n* = 24/Groups), haematoxylin and eosin and Masson's Trichrome were performed in sections to quantify the ratio of infarct size and collagen fibre. The sections were stained with haematoxylin and eosin and Masson's Trichrome. Details were described in supplementary materials.

### Immunofluorescence staining

To investigate the survival of ADSCs, the sections were stained with an anti-cTnI antibody. Cell survival was evaluated by identification of mRFP expression in confocal laser microscopic images. The numbers of mRFP^+^ cells and DAPI in each slide were calculated. The data were expressed as the percentage of mRFP^+^/DAPI. Vascular density was determined by immunofluorescent staining for vWF. The differentiation of ADSCs in the infarcted heart was identified by immunofluorescent staining for cardiac troponin T (cTnT) and α-smooth muscle actin (SMA), the differentiation rates of ADSCs were also calculated (detailed in the supplementary material).

### *In vitro* BLI assays

To evaluate the effect of Exendin-4 on the protection of ADSCs from ischaemic injury, the H_2_O_2_/SD was used to mimic the ischaemic environments. The fluc/mRFP positive ADSCs were plated in 48-well plates (2 × 10^4^ cells/well). After 24 hrs, ADSCs were given normal medium or normal medium plus Exendin-4. Twelve hours later, culture medium was removed. Cells were given serum deprivation plus H_2_O_2_ for 12 hrs. Cells without any treatment were used as control. After incubation with the reporter probe D-luciferin, ADSCs bioluminescence imaging was detected by using the Xenogen optical marcroscopic imaging system (Caliper Life Sciences, Hopkinton, MA, USA). Bioluminescence signals were measured by using Living Image 4.0 software (Caliper Life Sciences) and quantified with unit of photons/sec/cm^2^/sr.

### Detection of ADSCs apoptosis

The apoptosis of ADSCs was measured by using an Annexin V/PI Assay Kit (Biolegend, San Diego, CA, USA) according to the manufacturer's instructions. The percentages of apoptotic cells were analysed by flow cytometry (BD Biosciences, Franklin Lakes, NJ, USA). Caspase-3 activity was detected by using a Caspase-3 Activity Assay kit (Cell Signaling, Beverly, MA, USA) according to the manufacturer's instructions.

### ELISA

Adipose-derived stem cells were collected after being incubated with or without Exendin-4 for 24 hrs. The concentrations of paracrine factors from cells, including VEGF, HGF, bFGF and IGF-1, were measured by ELISA (VEGF–Sigma-Aldrich; HGF–B-Bridge (Mountian View, CA, USA); bFGF–Biotang (Waltham, MA, USA); IGF-1–Biotang) kits according to the manufacturer's instructions.

### Quantitative real-time polymerase chain reaction (qPCR)

Adipose-derived stem cells treated with or without Exendin-4 for 24 hrs were collected. Total RNA was isolated from cells by using TRIzol Reagent (Ambion, Carlsbad, CA, USA). First-strand cDNA was synthesized by using Thermo First cDNA Synthesis Kit (Germany) according to the standard procedures. The qPCRs were performed in triplicate with the FastStart Universal SYBR Green Master (ROX; Roche, Mannheim, Germany) and run on the StepOnePLUS system (Applied Biosystems, Foster City, CA, USA). The results were obtained from three independent experiments, whereby a no-template control was included. All primers were designed by the Primer 5 software (Premier Biosoft International, Palo Alto, Canada) and listed in the supplementary data.

### Western blotting

Protein samples were extracted from whole heart homogenates (*n* = 6/group, chosen randomly) and ADSCs as previously described [21]. Primary antibody against tumour necrosis factor-α (TNF-α) (R&D, Minneapolis, MN, USA), Akt1/2/3 (Santa Cruze, Santa Cruz, CA, USA), phospho-Akt (Santa Cruze), ERK (Cell Signaling), phosphor-ERK (Cell Signaling), signal transducers and activators of transcription 3 (STAT3; Cell Signaling), phosphor- STAT3 (Cell Signaling), Bcl-2 (Santa Cruze), Bax (Santa Cruze) and horseradish peroxidase–conjugated secondary antibody (Cell Signaling Technology) were used according to manufacturers’ instructions. GAPDH and β-actin antibody (Cell Signaling Technology) was used to evaluate the amount of protein loaded in each sample (detailed in the supplementary material).

### Statistics

Data are presented as mean ± SD. Statistical analyses were performed with SPSS software (version 17.0). Statistical significance between two groups was determined by Student's *t*-test. Results for more than two groups were evaluated by one-way anova with least significant difference test. *P* < 0.05 was considered as significant difference.

## Results

### Expansion and characterization of ADSCs

Our previous study has confirmed that fibroblast-like adherent cells derived from adipose tissue were multipotent, characterized by their ability to differentiate into adipocytes and osteoblasts [21,22,28]. After sorting by FACS, the percentage of mRFP-positive cells was up to 97.95% (Fig. S1D). After three passages, most of adherent ADSCs expressed stromal marker CD29 and CD90. In contrast, they seldom exhibited expression of haematopoietic marker CD34 and CD45. They were also negative for vascular endothelial cell marker CD31 (Fig. S1A). These ADSCs were multipotent as judged by their ability to differentiate into adipocytes, osteoblasts (Fig. S1B and C). BLI indicated a strong linear correlation between the cell number of ADSCs and the intensity of fluc signals (*R*^2^ = 0.997; Fig. S1E). The results confirmed that the luciferase reporter could be competent for quantifying the retention of transplanted ADSCs.

### Effect of Exendin-4 on myocardial ROS production

**Fig. 1:**
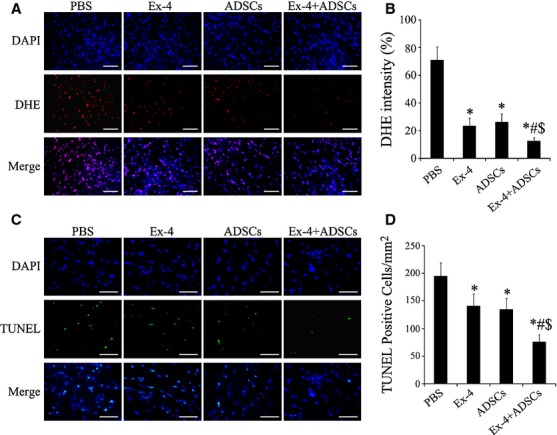
Myocardial reactive oxygen species generation and apoptosis at 1 week after myocardial infarction. (**A**) Dihydroethidium (DHE) staining showed a least red fluorescence intensity in combination-treated group compared with the other groups. (**B**) Quantification analysis for DHE staining (*n* = 6). (**C**) Representative images of TUNEL staining for groups. (**D**) Quantification analysis for TUNEL staining (*n* = 6); scale bars = 50 μm. *P < 0.05 *versus* PBS group; #*P* < 0.05 *versus* Exendin-4 group; $*P* < 0.05 *versus* adipose-derived stem cell group.

Reactive oxygen species is the critical mediator of cardiac injury and protection, while Exendin-4 treatment could act as an effective scavenger of ROS because of its attenuation of DHE staining (Fig. 1A). Signal intensity in Exendin-4 or ADSCs group is significantly lower than that in control group at day 7 after transplantation (*P* < 0.05, respectively; Fig. 1B). Notably, the level of ROS in combined group was the lowest among groups (*P* < 0.05 respectively). The result exhibited the antioxidant property of Exendin-4 as well as an antioxidant synergism in the combination of Exendin-4 and ADSCs treatment.

### Apoptosis in the infarcted heart

At 1 week after surgery, TUNEL-positive cells in the Exendin-4 or ADSCs group were significantly lower than those in control group (*P* < 0.05 respectively). The TUNEL-positive cells in Exendin-4+ ADSCs group were the lowest among all groups (*P* < 0.05 respectively; Fig. 1C and D). These data suggested an anti-apoptotic synergism in the combination of Exendin-4 and ADSCs treatment.

### Bioluminescence imaging

**Fig. 2:**
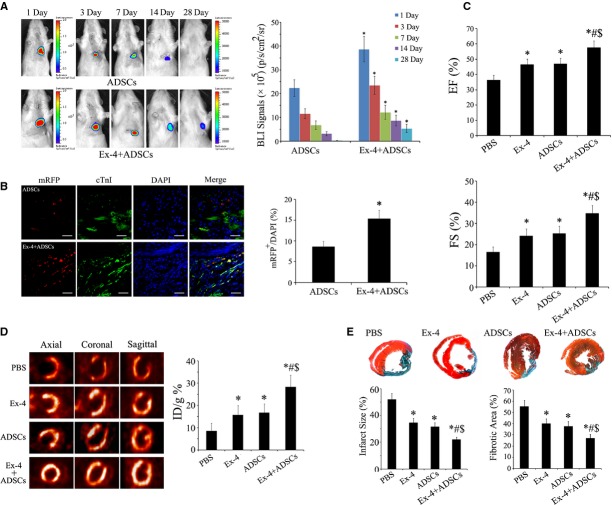
The therapeutic efficacy of adipose-derived stem cells (ADSCs) adjuvant with exendin-4 for myocardial infarction. (**A**) Representative BLI images of rat and quantitative analysis from ADSCs group and Exendin-4+ ADSCs group. *P < 0.05 *versus* ADSCs group; *n* = 12. (**B**) Confocal laser microscopic images and quantitative analysis of the ratio of mRFP/DAPI cells at 4 weeks after transplantation; scale bars = 50 μm. *P < 0.05; *n* = 6. (**C**) Echocardiography showed that EF and FS were significantly improved in rats treated with Exendin-4+ ADSCs group, *n* = 12. (**D**) Representative images of Micro-PET in infarcted hearts among groups and quantitative analysis, *n* = 5. (**E**) Representative images of Masson trichrome staining and statistical results of infarct size and fibrotic area, *n* = 24. *P < 0.05 *versus* PBS group; #*P* < 0.05 *versus* Exendin-4 group; $*P* < 0.05 *versus* ADSCs group.

The injection of ADSCs led to a strong bioluminescence signal at day 1. The BLI signals in the Exendin-4+ ADSCs group were about 1.5 times of that in ADSCs group (*P* < 0.05), suggesting that Exendin-4 effectively enhanced the retention of transplanted ADSCs compared with Exendin-4 untreated group. During the initial 3–14 days, serial imaging of the same rats confirmed a gradually decreased bioluminescence signal. However, the BLI signal intensity of Exendin-4+ ADSCs group is constantly stronger than that of ADSCs group. At day 28, nearly no signals could be detected in ADSCs group, indicating a great loss of engrafted ADSCs. In comparison, modest signals were still detected in combined group (Fig. 2A). These results demonstrated that Exendin-4 treatment could significantly improve the retention and survival of injected ADSCs in ischaemic myocardium.

To confirm *in vivo* BLI results for ADSCs survival, cell survival was further evaluated by calculating the ratio of mRFP^+^/DAPI at 4 weeks after transplantation. More mRFP^+^ cells could be observed in the Exendin-4+ ADSCs group (Fig. 2B). The ratio of mRFP^+^/DAPI in Exendin-4+ ADSCs group was 15.3 ± 2.1%, significantly higher than that in the ADSCs only group (8.6 ± 1.3%, *P* < 0.05). These results demonstrated that Exendin-4 could significantly improve the survival of injected ADSCs in ischaemic myocardium.

### Cardiac function

Four weeks after transplantation, EF and FS were significantly improved in Exendin-4, ADSCs and Exendin-4+ ADSCs group compared with PBS group, with the best LV function in Exendin-4+ ADSCs group (Fig. 2C; *P* < 0.05). A synergism occurred in combination with Exendin-4 and ADSCs, which markedly improved the LV parameters.

### Assessment of myocardial activity

[^18^F]-fluorodeoxyglucose is most widely used in PET measurement, the uptake of which reflects the changes in myocardial viability followed by myocardial ischaemia. We performed micro-PET scans of rats with [^18^F]-FDG at day 28 following infarction (Fig. 2D). The myocardial uptake of [^18^F]-FDG increased significantly in all of the three treated groups compared with the PBS group (*P* < 0.05 respectively). There was no significant difference between the Exendin-4 only group and ADSCs only group (*P* > 0.05).

### Myocardial infarct size and collagen deposition

Masson's Trichrome staining showed a marked reduction in left ventricle fibrosis as observed in treatment group compared with PBS group (*P* < 0.05, respectively; Fig. 2E). Collagen deposition in the infarcted myocardium was much less in all of three treatment groups than that in the PBS group. The percentage of fibrotic area was significantly lower in the Exendin-4 or ADSCs or Exendin-4+ ADSCs groups compared with that in the PBS group (*P* < 0.05 respectively). The least collagen deposition was observed in the Exendin-4+ ADSCs group among groups (*P* < 0.05 respectively).

### Differentiation of ADSCs in infarcted myocardium

**Fig. 3:**
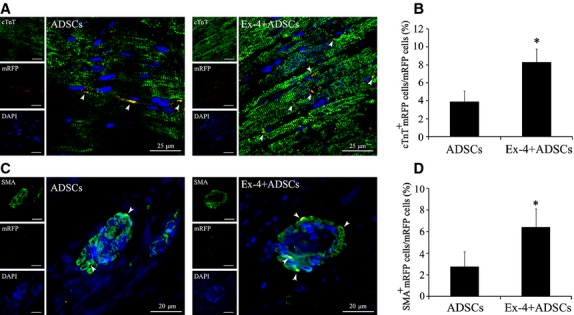
Confocal laser microscopic images for adipose-derived stem cells (ADSCs) differentiation in the peri-infarct area. (**A**) Representative immunofluorescence images for cTnT+/mRFP+ cardiomyocytes. (**B**) Quantification analysis of cardiomyocyte differentiation of ADSCs. *n* = 12. (**C**) Representative immunofluorescence images for α-SMA+/mRFP+ vascular smooth muscle cells. (**D**) Quantification analysis of vascular smooth muscle cells differentiation of ADSCs. *n* = 12; scale bars = 30 μm. *P < 0.05 *versus* ADSCs group.

Immunofluorescent analysis of cardiac- and blood vessel–specific proteins in both ADSCs and Ex-4+ ADSCs groups indicated that the hearts with Ex-4+ ADSCs treatment contained an increased number of cells carrying the differentiation markers for cardiomyocytes (cTnT+/mRFP+, Fig. 3A) and vascular smooth muscle cells (SMA+/mRFP+, Fig. 3C) compared with that in the animals treated with ADSCs alone. The percentage of cTnT+/mRFP+ cells was much higher in the Ex-4+ ADSCs group compared with that in the ADSCs group (8.26 ± 1.53% *versus* 3.89 ± 1.21%, *P* < 0.05, Fig. 3B). Connexin43-positive cells derived from ADSCs were also observed in the Exendin-4+ ADSCs treated group (Fig. S3), suggesting a structural and functional coupling. The percentage of SMA+/mRFP+ cells in the Ex-4+ ADSCs group was significantly higher than that in the ADSCs group (6.42 ± 1.71% *versus* 2.77 ± 1.36%, *P* < 0.05, Fig. 3D). We also found that the vascular density in Exendin-4+ ADSCs group was the highest among all groups (Fig. S4).

### ADSCs adjuvant with Exendin-4 leads to myocardial activation of STAT3

**Fig. 4:**
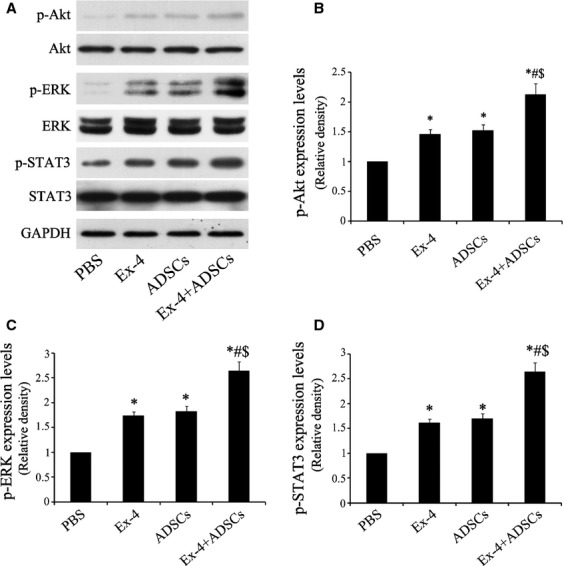
Adipose-derived stem cells (ADSCs) adjuvant with Exendin-4 activated the Akt, ERK1/2 and STAT3 in ischaemic myocardium. (**A**) Representative western blotting of p-Akt, Akt, p-ERK, ERK, p-STAT3, STAT3 and GAPDH in ischaemic myocardium. (**B**–**D**) The relative optical density ratio of p-Akt, p-ERK, p-STAT3 (*n* = 6). *P < 0.05 *versus* PBS group; #*P* < 0.05 *versus* Exendin-4 group; $*P* < 0.05 *versus* ADSCs group.

To gain insight into the mechanism involved in the protective effect of combined therapy on ischaemic myocardium, we examined the phosphorylation of Akt, ERK1/2 and STAT3 by western blotting (Fig. 4A). The levels of p-Akt, p-ERK1/2 were the highest in the combined group compared with other groups (Fig. 4B and C, *P* < 0.05). Furthermore, the expression level of STAT3 in Ex-4+ ADSCs group increased significantly (Fig. 4D, *P* < 0.05).

### Exendin-4 protects ADSCs from H_2_O_2_/SD-induced apoptosis *via* activation of STAT3

**Fig. 5:**
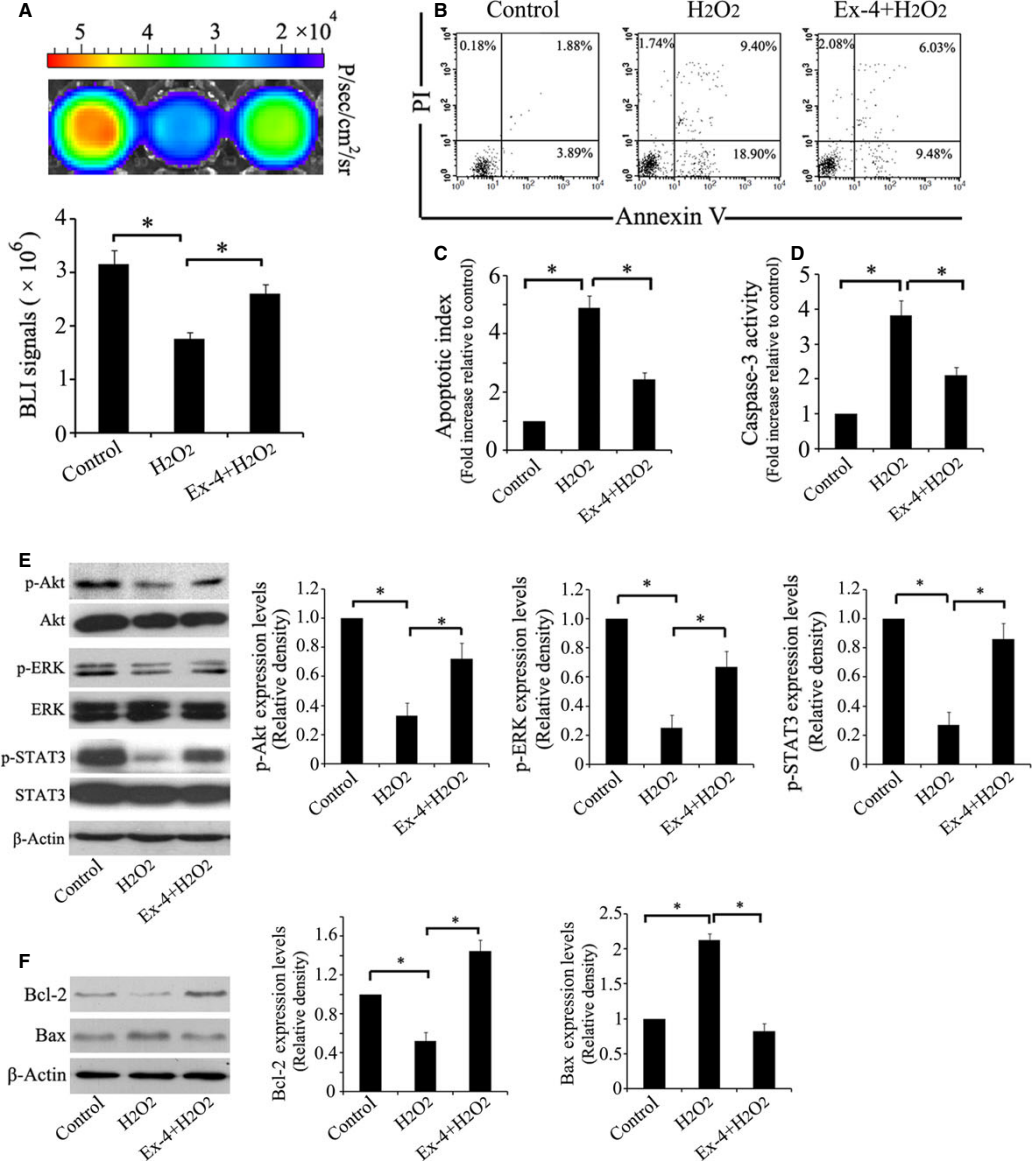
Exendin-4 prevented H_2_O_2_/SD- induced apoptosis of adipose-derived stem cells (ADSCs) and activated Akt, ERK and STAT3. (**A**) In vitro BLI determined that Exendin-4 at 5 nM promoted the attenuated viability of ADSCs exposed to H_2_O_2_/SD (*n* = 6). (**B**) Apoptosis was evaluated with flow cytometry analysis. (**C**) The quantitative analysis of apoptotic index (apoptotic cells/total cells) in ADSCs (*n* = 6). (**D**) Caspase-3 activity determined by using Caspase-3 ELISA kit (*n* = 6). (**E**) Representative blots of p-Akt, Akt, p-ERK, ERK, p-STAT3, STAT3 in ADSCs exposed to H_2_O_2_/SD and quantitative analysis of p-Akt, p-ERK, p-STAT3. β-actin is used as internal parameter (*n* = 6). (**F**) Representative blots of Bcl-2 and Bax in ADSCs exposed to H_2_O_2_/SD and quantitative analysis of Bcl-2 and Bax (*n* = 6); *P < 0.05.

Adipose-derived stem cells were pre-treated with Exendin-4 at concentrations of 0.001, 0.01, 0.1, 1, 5, 10 nM for 12 hrs. Then they were treated by 100 μM H_2_O_2_/SD for another 12 hrs. Flow cytometry showed that the optimal anti-apoptotic concentration of Exendin-4 was 5 nM (data not shown). *In vitro* BLI demonstrated that H_2_O_2_/SD injury for 12 hrs impaired the viability of ADSCs while 5 nM Exendin-4 improved the viability of ADSCs significantly (*P* < 0.05, Fig. 5A). Furthermore, Exendin-4 decreased H_2_O_2_/SD-induced apoptosis of ADSCs significantly (*P* < 0.05, Fig. 5B and C). We also found that Exendin-4 significantly reduced caspase-3 activity in ADSCs induced by H_2_O_2_/SD (*P* < 0.05, Fig. 5D). These results suggest that Exendin-4 decreases H_2_O_2_/SD-induced apoptosis of ADSCs.

To understand the possible mechanisms in Exendin-4-mediated anti-apoptotic effects, we examined p-Akt/Akt, p-ERK/ERK, p-STAT3/STAT3 activity by using Western blotting (Fig. 5E). Exendin-4 treatment increased the levels of p-Akt, p-ERK, p-STAT3 significantly in ADSCs exposed to H_2_O_2_/SD (*P* < 0.05). To further evaluate the anti-apoptotic effects of Exendin-4, ADSCs were examined for anti-apoptotic protein Bcl-2 and pro-apoptotic protein Bax (Fig. 5F). The results showed that H_2_O_2_/SD significantly increased the level of Bax, but decreased the level of Bcl-2, which was rescued by Exendin-4 treatment (*P* < 0.05). Together, these results indicated that the protective effect of Exendin-4 may be related to STAT3 activation mediated by the phosphorylation of Akt and ERK1/2.

### Increased soluble factors

**Fig. 6:**
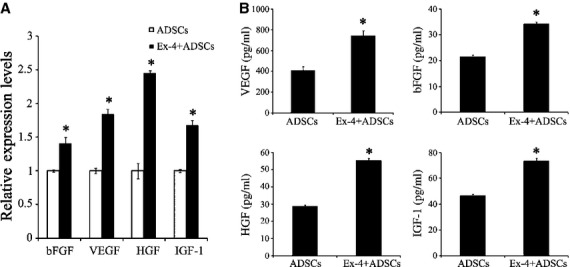
Exendin-4 increased paracrine factor release from adipose-derived stem cells (ADSCs). (**A**) Quantitative real-time PCR analysis of gene expression of VEGF, bFGF, HGF, IGF-1 in ADSCs with or without Exendin-4 treatment. (**B**) ELISA detection of VEGF, bFGF, HGF, IGF-1 levels in the ADSCs-conditioned medium with or without Exendin-4 treatment (*n* = 6); *P < 0.05 *versus* control.

The gene expression level of VEGF, bFGF, HGF, IGF-1 measured by qPCR showed increased mRNA expression in Exendin-4 pre-treated ADSCs than that in non-treated ADSCs (Fig. 6A). Notably, significant amounts of VEGF, bFGF, HGF, IGF-1 were released from Exendin-4 pre-treated ADSCs when compared with non-treated ADSCs (Fig. 6B).

## Discussion

Ischaemic milieu plays the pivotal role in the survival of exogenous stem cells in injured myocardium. It has been reported that more than 70–80% cells die within 3 days after transplantation [29]. Thus, it is imperative to find strategies to improve hostile environment to enhance graft cell survival. In this study, we demonstrated that Exendin-4 enhanced the survival and viability of ADSCs in infarcted rat hearts, which contributed to an improved cardioprotective effect of ADSCs. In addition, our data showed that Exendin-4 exerts its protective effect on ADSCs *via* the STAT3 activation mediated by the Akt and ERK1/2.

Experimental and clinical studies have clearly demonstrated the potential cardioprotective effect of GLP-1 and its analogue [13,14,30]. Glucagon-like peptide-1 is shown to protect stem cells from apoptosis *in vitro* [20]. To clarify whether Exendin-4 could serve as an adjuvant agent in stem cell therapy for the treatment of MI, we measured the *in vivo* BLI signals of ADSCs with or without Exendin-4 in the rat model of MI. The results indicated that the Exendin-4 enhanced the survival of transplanted ADSCs, demonstrated by the increased BLI signals. Furthermore, our results demonstrated that combined therapy of Exendin-4 and ADSCs significantly decreased fibrosis, improved LV function and myocardial activity (Fig. 2). The results indicate that the ADSCs transplantation adjuvant with Exendin-4 provided a promising strategy for stem cell–based therapy for MI.

There are several mechanisms underlying the benefit of ADSCs transplantation adjuvant with Exendin-4 for the recovery of the ischaemic tissue. Firstly, Exendin-4 promoted the expression and release of antioxidant factors of ADSCs, such as HGF and IGF-1. It is responsible for the decreased ROS levels in ischaemic myocardium (Fig. 1). As a consequence, ADSCs adjuvant with Exendin-4 significantly decreased inflammatory cell infiltration and down-regulated the expression of TNF-α in the peri-infarct area (Fig. S2). Secondly, increased secretion of angiogenic growth factors from ADSCs by Exendin-4, including VEGF and bFGF, potentially induced endothelial growth, migration and differentiation [31–33]. In this study, vessel density of peri-infarct area is highest in Exendin-4+ ADSCs group (Fig. S4). The angiogenesis of MI plays an important role in the survival and function of transplanted stem cells. Despite the paracrine effect, ADSCs may participate in the angiogenesis directly by differentiation into vascular smooth muscle cells as their rate of differentiation was higher in combined group (Fig. 3C). Thirdly, stem cell differentiation for the replacement of damaged tissues was conventionally regarded as the center of stem cell therapies for tissue regeneration. This study showed that Exendin-4 enhanced the differentiation of transplanted ADSCs into cardiomyocytes (Fig. 3A). Reportedly, ADSCs hold the ability to engraft injured myocardium. This engraftment was associated with expression of cardiomyocytic markers by donor-derived cells [34–36]. In this study, histological results show that the number of cardiomyocytes derived from ADSCs is much more in the combined group than that in the ADSCs group. Connexin43 was also observed in combined group (Fig. S3), suggesting the coupling of structure and function. In spite of the increased survival of transplanted ADSCs, these data indicated that Exendin-4 could promote cellular maturation and differentiation in ischaemic myocardium. Reportedly, GLP-1 agonists could influence the stem cell differentiation. For example, GLP-1 can mediate differentiation of human iPS cells and human ADSCs into insulin-secreting cells [37,38].

To better understand the mechanism of the benefits of Exendin-4 on ADSCs, we used H_2_O_2_/SD to mimic *in vivo* ischaemic insult (Fig. 5). Our *in vitro* BLI analysis showed that Exendin-4 could protect ADSCs from the injury of H_2_O_2_/SD. Moreover, Exendin-4 decreases apoptosis of ADSCs induced by H_2_O_2_/SD injury. The results from western blotting suggested that Exendin-4 promoted the functional survival of ADSCs *via* STAT3 activation through Akt and ERK1/2. Signal transducers and activators of transcription factors are a family of cytoplasmic transcription factors that mediate intracellular signalling initiated at cytokine cell surface receptors and transmitted to the nucleus [39]. Signal transducers and activators of transcription 3 plays pivotal roles in many cellular processes such as cell apoptosis by mediating the expression of a variety of genes under stressful conditions [40–42]. Studies have also demonstrated that kinases, such as Akt and ERK1/2, could phosphorylate STAT3 [43,44]. In addition, it is well established that the signalling pathways Akt, ERK and STAT3 are involved in protecting cells against injury and promoting cell survival [45]. Consistently, our results indicated that STAT3 could be activated through the phosphorylation of Akt and ERK1/2 both in ADSCs and myocardium. Furthermore, we demonstrated that Exendin-4 treatment increased the levels of the anti-apoptotic protein Bcl-2 while decreased pro-apoptotic protein Bax in ADSCS. Reportedly, both Bcl-2 and Bax were the downstream targets of STAT3 [42,46]. In addition, multiple studies have suggested that the STAT3 was linked to a protective and reparative response in the myocardium. Increasing duration or intensity of STAT3 activation could minimize injuries and improve cardiac function under conditions of stress [39,40,45]. In this study, we showed that the benefits of ADSCs adjuvant with Exendin-4 facilitating cardiac repairs were mediated by the activation of STAT3. Nevertheless, STAT3 may also hold the potential to modulate the paracrine effects of stem cell. It was reported that the STAT3 could mediate bone marrow mesenchymal stem cell VEGF production [47] in consistency with our findings that Exendin-4 promoted the release of VEGF as well as the STAT3 expression. This is a first attempt to elucidate the signals involved in Exendin-4-mediated protective properties in ADSCs and injured myocardium.

In conclusion, we have furnished evidence that Exendin-4 could effectively enhance the survival and viability of ADSCs transplanted in ischaemic myocardium. The ADSCs adjuvant with Exendin-4 may have a synergistic effect on cardiac performances. In addition, Exendin-4 could protect ADSCs against H_2_O_2_/SD-induced injury through STAT3 activation *via* the phosphorylation of Akt and ERK1/2. This study provides a novel candidate method for the stem cell–based therapies of MI patients.

## Supporting information

**Figure S1** Characterization of lentiviral transduced ADSCs expressing fluc-mRFP dual fusion reporter genes. **Figure S2** Inflammatory response at 4 weeks after transplantation. **Figure S3** Connexin43 (Cx43) expression of ADSCs derived mRFP+ cells in the peri-infarct area. **Figure S4** Vascular density in the peri-infarct area.

## References

[1] Shah VK, Shalia KK (2011). Stem cell therapy in acute myocardial infarction: a pot of gold or Pandora's box. Stem Cells Int.

[2] Kikuchi K, Poss KD (2012). Cardiac regenerative capacity and mechanisms. Annu Rev Cell Dev Biol.

[3] Segers VF, Lee RT (2008). Stem-cell therapy for cardiac disease. Nature.

[4] Mias C, Lairez O, Trouche E (2009). Mesenchymal stem cells promote matrix metalloproteinase secretion by cardiac fibroblasts and reduce cardiac ventricular fibrosis after myocardial infarction. Stem Cells.

[5] Kuethe F, Richartz BM, Kasper C (2005). Autologous intracoronary mononuclear bone marrow cell transplantation in chronic ischemic cardiomyopathy in humans. Int J Cardiol.

[6] Bai X, Yan Y, Song YH (2010). Both cultured and freshly isolated adipose tissue-derived stem cells enhance cardiac function after acute myocardial infarction. Eur Heart J.

[7] Gimble JM, Katz AJ, Bunnell BA (2007). Adipose-derived stem cells for regenerative medicine. Circ Res.

[8] Stephan MT, Irvine DJ (2011). Enhancing cell therapies from the outside in: cell surface engineering using synthetic nanomaterials. Nano Today.

[9] Yang YJ, Qian HY, Huang J (2009). Combined therapy with simvastatin and bone marrow-derived mesenchymal stem cells increases benefits in infarcted swine hearts. Arterioscler Thromb Vasc Biol.

[10] Yang YJ, Qian HY, Huang J (2008). Atorvastatin treatment improves survival and effects of implanted mesenchymal stem cells in post-infarct swine hearts. Eur Heart J.

[11] Khan M, Meduru S, Mohan IK (2009). Hyperbaric oxygenation enhances transplanted cell graft and functional recovery in the infarct heart. J Mol Cell Cardiol.

[12] Khan M, Meduru S, Gogna R (2012). Oxygen cycling in conjunction with stem cell transplantation induces NOS3 expression leading to attenuation of fibrosis and improved cardiac function. Cardiovasc Res.

[13] Ban K, Noyan-Ashraf MH, Hoefer J (2008). Cardioprotective and vasodilatory actions of glucagon-like peptide 1 receptor are mediated through both glucagon-like peptide 1 receptor-dependent and -independent pathways. Circulation.

[14] Anagnostis P, Athyros VG, Adamidou F (2011). Glucagon-like peptide-1-based therapies and cardiovascular disease: looking beyond glycaemic control. Diabetes Obes Metab.

[15] Kodera R, Shikata K, Kataoka HU (2011). Glucagon-like peptide-1 receptor agonist ameliorates renal injury through its anti-inflammatory action without lowering blood glucose level in a rat model of type 1 diabetes. Diabetologia.

[16] Bunck MC, Corner A, Eliasson B (2010). One-year treatment with exenatide *vs*. insulin glargine: effects on postprandial glycemia, lipid profiles, and oxidative stress. Atherosclerosis.

[17] Mukai E, Fujimoto S, Sato H (2011). Exendin-4 suppresses SRC activation and reactive oxygen species production in diabetic Goto-Kakizaki rat islets in an Epac-dependent manner. Diabetes.

[18] Velmurugan K, Balamurugan AN, Loganathan G (2012). Antiapoptotic actions of exendin-4 against hypoxia and cytokines are augmented by CREB. Endocrinology.

[19] Oeseburg H, de Boer RA, Buikema H (2010). Glucagon-like peptide 1 prevents reactive oxygen species-induced endothelial cell senescence through the activation of protein kinase A. Arterioscler Thromb Vasc Biol.

[20] Sanz C, Vazquez P, Blazquez C (2010). Signaling and biological effects of glucagon-like peptide 1 on the differentiation of mesenchymal stem cells from human bone marrow. Am J Physiol Endocrinol Metab.

[21] Liu Z, Wang H, Wang Y (2012). The influence of chitosan hydrogel on stem cell engraftment, survival and homing in the ischemic myocardial microenvironment. Biomaterials.

[22] Zhang X, Wang H, Ma X (2010). Preservation of the cardiac function in infarcted rat hearts by the transplantation of adipose-derived stem cells with injectable fibrin scaffolds. Exp Biol Med.

[23] Cao F, Lin S, Xie X (2006). In vivo visualization of embryonic stem cell survival, proliferation, and migration after cardiac delivery. Circulation.

[24] Brown SB, Libonati JR, Selak MA (2010). Neonatal exendin-4 leads to protection from reperfusion injury and reduced rates of oxidative phosphorylation in the adult rat heart. Cardiovasc Drugs Ther.

[25] Khan M, Kutala VK, Vikram DS (2007). Skeletal myoblasts transplanted in the ischemic myocardium enhance in situ oxygenation and recovery of contractile function. Am J Physiol Heart Circ Physiol.

[26] Cao Q, Li ZB, Chen K (2008). Evaluation of biodistribution and anti-tumor effect of a dimeric RGD peptide-paclitaxel conjugate in mice with breast cancer. Eur J Nucl Med Mol Imaging.

[27] Li Z, Lee A, Huang M (2009). Imaging survival and function of transplanted cardiac resident stem cells. J Am Coll Cardiol.

[28] Gao J, Liu R, Wu J (2012). The use of chitosan based hydrogel for enhancing the therapeutic benefits of adipose-derived MSCs for acute kidney injury. Biomaterials.

[29] Haider H, Ashraf M (2008). Strategies to promote donor cell survival: combining preconditioning approach with stem cell transplantation. J Mol Cell Cardiol.

[30] Nikolaidis LA, Mankad S, Sokos GG (2004). Effects of glucagon-like peptide-1 in patients with acute myocardial infarction and left ventricular dysfunction after successful reperfusion. Circulation.

[31] Rehman J, Traktuev D, Li J (2004). Secretion of angiogenic and antiapoptotic factors by human adipose stromal cells. Circulation.

[32] Moon MH, Kim SY, Kim YJ (2006). Human adipose tissue-derived mesenchymal stem cells improve postnatal neovascularization in a mouse model of hindlimb ischemia. Cell Physiol Biochem.

[33] Mazo M, Planat-Benard V, Abizanda G (2008). Transplantation of adipose derived stromal cells is associated with functional improvement in a rat model of chronic myocardial infarction. Eur J Heart Fail.

[34] Strem BM, Zhu M, Alfonso Z (2005). Expression of cardiomyocytic markers on adipose tissue-derived cells in a murine model of acute myocardial injury. Cytotherapy.

[35] Planat-Benard V, Menard C, Andre M (2004). Spontaneous cardiomyocyte differentiation from adipose tissue stroma cells. Circ Res.

[36] Jumabay M, Zhang R, Yao Y (2010). Spontaneously beating cardiomyocytes derived from white mature adipocytes. Cardiovasc Res.

[37] Thatava T, Nelson TJ, Edukulla R (2011). Indolactam V/GLP-1-mediated differentiation of human iPS cells into glucose-responsive insulin-secreting progeny. Gene Ther.

[38] Wei AH, Wang WJ, Mu XP (2012). Enhanced differentiation of human adipose tissue-derived stromal cells into insulin-producing cells with glucagon-like peptide-1. Exp Clin Endocrinol Diabetes.

[39] Suleman N, Somers S, Smith R (2008). Dual activation of STAT-3 and Akt is required during the trigger phase of ischaemic preconditioning. Cardiovasc Res.

[40] Negoro S, Kunisada K, Fujio Y (2001). Activation of signal transducer and activator of transcription 3 protects cardiomyocytes from hypoxia/reoxygenation-induced oxidative stress through the upregulation of manganese superoxide dismutase. Circulation.

[41] Barry SP, Townsend PA, McCormick J (2009). STAT3 deletion sensitizes cells to oxidative stress. Biochem Biophys Res Commun.

[42] Xu Y, Ikegami M, Wang Y (2007). Gene expression and biological processes influenced by deletion of Stat3 in pulmonary type II epithelial cells. BMC Genomics.

[43] Zhang X, Shan P, Alam J (2005). Carbon monoxide differentially modulates STAT1 and STAT3 and inhibits apoptosis *via* a phosphatidylinositol 3-kinase/Akt and p38 kinase-dependent STAT3 pathway during anoxia-reoxygenation injury. J Biol Chem.

[44] Chung J, Uchida E, Grammer TC (1997). STAT3 serine phosphorylation by ERK-dependent and -independent pathways negatively modulates its tyrosine phosphorylation. Mol Cell Biol.

[45] Huang C, Gu H, Zhang W (2011). SDF-1/CXCR4 mediates acute protection of cardiac function through myocardial STAT3 signaling following global ischemia/reperfusion injury. Am J Physiol Heart Circ Physiol.

[46] Liu H, Jiang C, Xiong C (2012). DEDC, a new flavonoid induces apoptosis *via* a ROS-dependent mechanism in human neuroblastoma SH-SY5Y cells. Toxicol In Vitro.

[47] Wang M, Zhang W, Crisostomo P (2007). STAT3 mediates bone marrow mesenchymal stem cell VEGF production. J Mol Cell Cardiol.

