# Supplementary material for: The stem cell adjuvant with Exendin-4 repairs the heart after myocardial infarction via STAT3 activation
Source: J Cell Mol Med. 2014 Apr 30;18(7):1381–91. doi: 10.1111/jcmm.12272 (PMC4124022; doi:10.1111/jcmm.12272)
Supplement: Supplementary file 1 [file jcmm0018-1381-SD1.docx]

**Supplemental Results**

**Effect of Exendin-4 on inflammation response**

At day 28 after transplantation, in the peri-infarct region, H&E staining demonstrated that inflammatory cell infiltration in Exendin-4 or ADSCs treated group is lower than that in control group (*P*<0.05, respectively), with the fewest inflammatory cell infiltration in Exendin-4+ADSCs group (Fig.S2A and B; *P*<0.05). The expression of the inflammatory protein TNF-α was significantly down-regulated in Exendin-4 treated group compared with untreated rats, especially in the combination group (Fig.S2C; *P*<0.05).

**Supplemental Figures and Figure Legends**

**
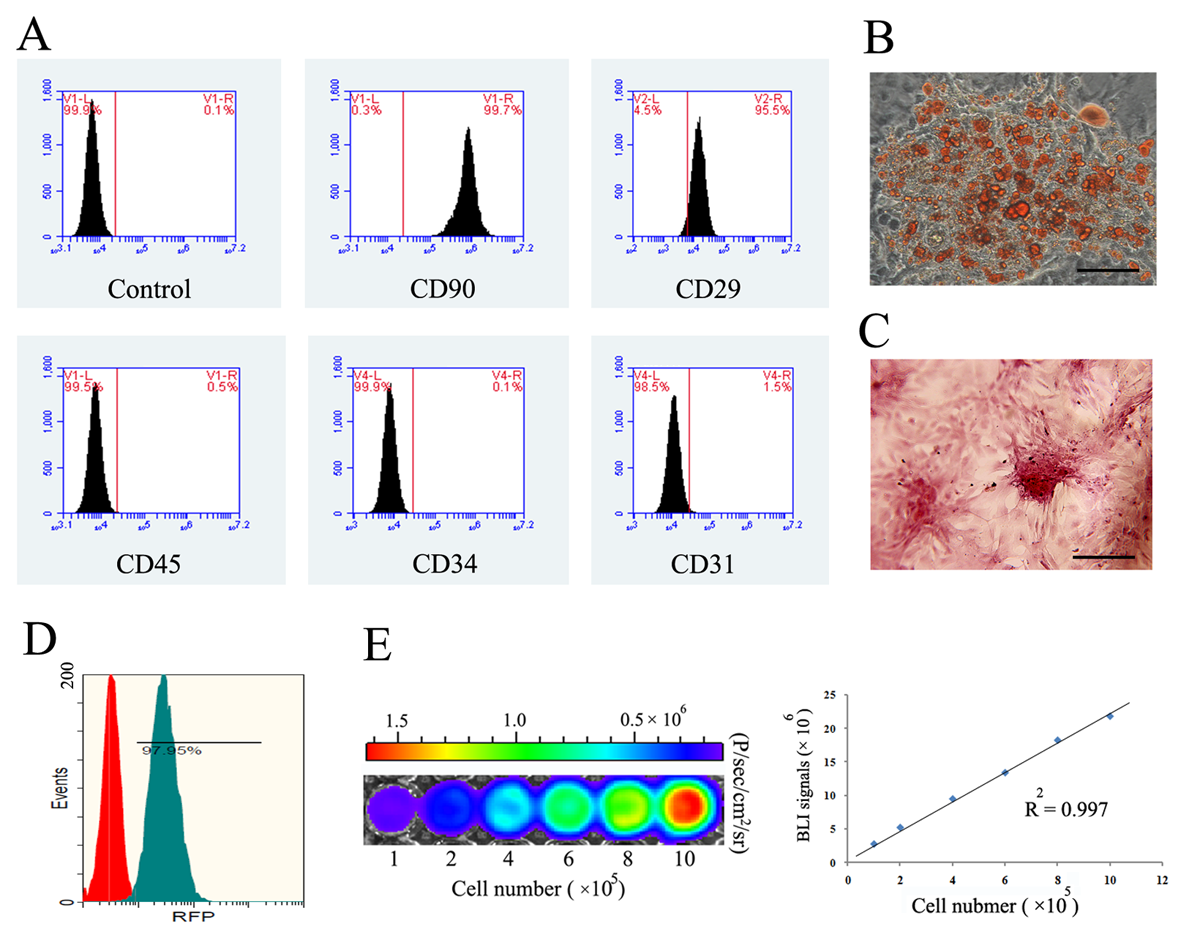
**

**FigureS1. Characterization of lentiviral transduced ADSCs expressing fluc-mRFP dual fusion reporter genes.** (A) Flow cytometric analysis of ADSCs was performed using CD29, CD90, CD31, CD34 and CD45 antibodies. (B and C) ADSCs labeled with fluc-mRFP reporter exhibit the potential to differentiate into adipocytes (B) and osteoblasts (C) Scale bars= 100μm. (D) Most of ADSCs expressed reporter after sorting. (E) Bioluminescence imaging showed BLI signal intensity was positive proportional to cell numbers.

**
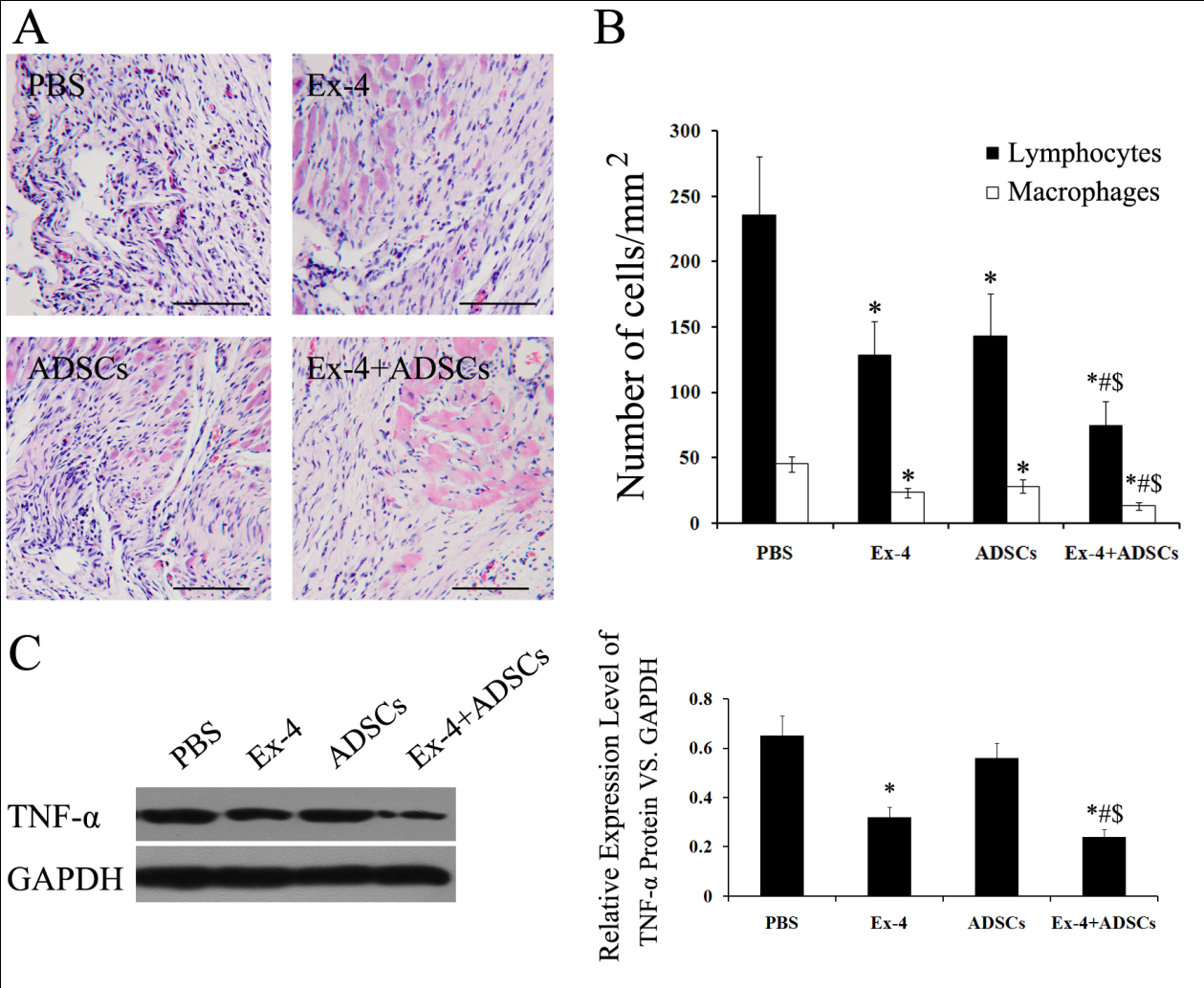
**

**FigureS2. Inflammatory response at 4 weeks after transplantation.** (A) H&E staining showed the inflammatory cell infiltration of different groups. (B) Quantitative analysis showed inflammatory cell number in Exendin-4+ADSCs group was significantly decreased than that in PBS group. (C) TNF-α was markedly attenuated in both Exendin-4 and Exendin-4+ADSCs group as measured by western blotting. GAPDH was used as an internal control parameter. Scale bar= 100μm. **P*<0.05 versus PBS group; # *P*<0.05 versus Exendin-4 group; $ *P*<0.05 versus ADSCs group.

**
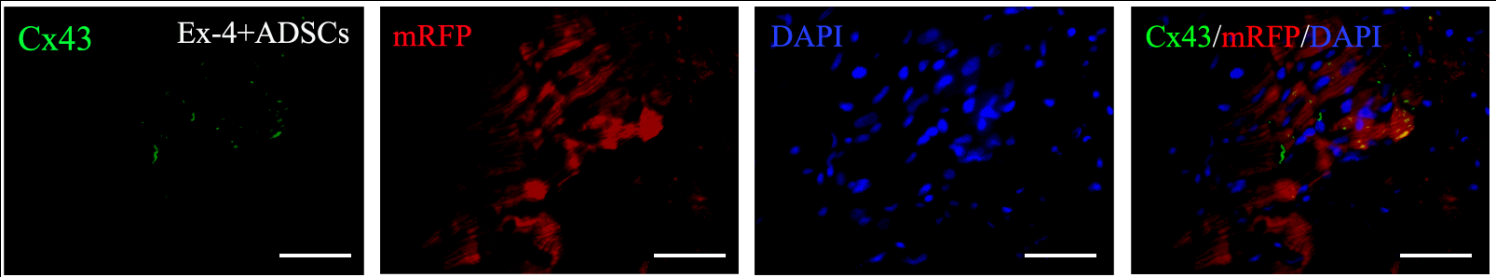
**

**Figure S3. Connexin43 (**Cx43**) expression of ADSCs derived mRFP+ cells in the peri-infarct area.** Immunofluorescence staining for connexin43 showed that connexin43 could be observed in the Exendin-4+ADSCs treated group. Hardly no Cx43+/mRFP+ cells could be found in ADSCs alone treatment group (data not shown). Scale bars= 30μm.

**
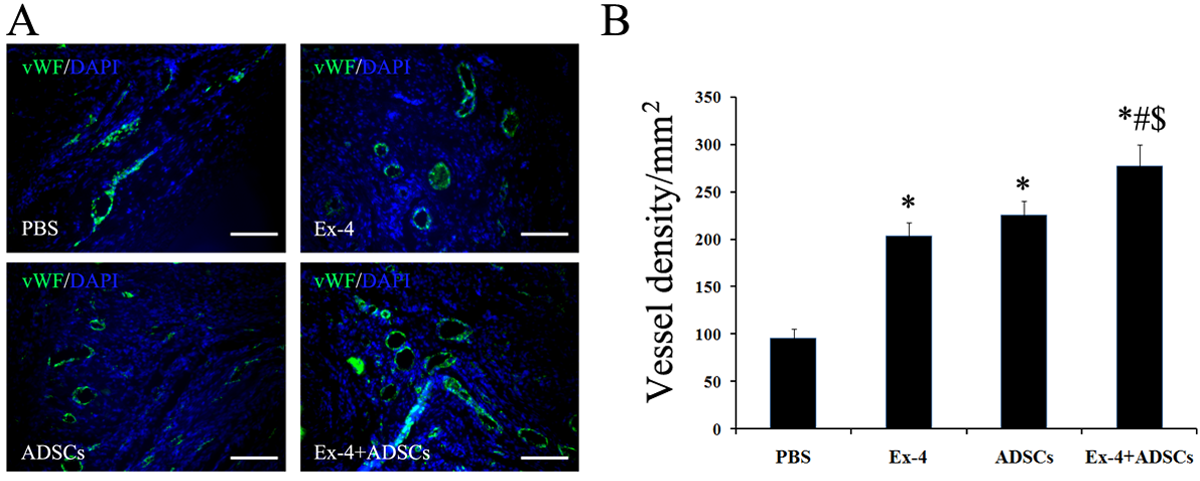
**

**Figure S4. Vascular density in the peri-infarct area.** (A) Immunofluorescence staining for vWF showed increasing positive microvessels in hearts from group Exendin-4, ADSCs, Exendin-4+ADSCs. (B) Quantification analysis of vWF positive microvessels revealed the vessel density in Exendin-4+ADSCs group is highest compared with those in other groups. Scale bars= 100μm. **P*<0.05 versus PBS group; # *P*<0.05 versus Exendin-4 group; $ *P*<0.05 versus ADSCs group.

**Supplemental references**

1. **Liu Z, Wang H, Wang Y, *et al*.** The influence of chitosan hydrogel on stem cell engraftment, survival and homing in the ischemic myocardial microenvironment. *Biomaterials*. 2012; 33: 3093-106.

2. **Zhang X, Wang H, Ma X, *et al*.** Preservation of the cardiac function in infarcted rat hearts by the transplantation of adipose-derived stem cells with injectable fibrin scaffolds. *Exp Biol Med (Maywood)*. 2010; 235: 1505-15.

3. **Cao F, Lin S, Xie X, *et al*.** In vivo visualization of embryonic stem cell survival, proliferation, and migration after cardiac delivery. *Circulation*. 2006; 113: 1005-14.

4. **Khan M, Meduru S, Gogna R, *et al*.** Oxygen cycling in conjunction with stem cell transplantation induces NOS3 expression leading to attenuation of fibrosis and improved cardiac function. *Cardiovasc Res*. 2012; 93: 89-99.

5. **Brown SB, Libonati JR, Selak MA, *et al*.** Neonatal exendin-4 leads to protection from reperfusion injury and reduced rates of oxidative phosphorylation in the adult rat heart. *Cardiovasc Drugs Ther*. 2010; 24: 197-205.

6. **Singelyn JM, Sundaramurthy P, Johnson TD, *et al*.** Catheter-deliverable hydrogel derived from decellularized ventricular extracellular matrix increases endogenous cardiomyocytes and preserves cardiac function post-myocardial infarction. *J Am Coll Cardiol*. 2012; 59: 751-63.

7. **Cavasin MA, Tao Z, Menon S, Yang XP.** Gender differences in cardiac function during early remodeling after acute myocardial infarction in mice. *Life Sci*. 2004; 75: 2181-92.
