# Supplementary material for: The stem cell adjuvant with Exendin-4 repairs the heart after myocardial infarction via STAT3 activation
Source: J Cell Mol Med. 2014 Apr 30;18(7):1381–91. doi: 10.1111/jcmm.12272 (PMC4124022; doi:10.1111/jcmm.12272)
Supplement: Supplementary file 2 — Data S1 Supplemental methods. [file jcmm0018-1381-SD2.doc]

**Supplementary Data**

**Manuscript title:** The Stem Cell Adjuvant with Exendin-4 Repair the Heart after Myocardial Infarction via STAT3 Activation

**Running title:** Exendin-4 facilitate ADSCs to treat MI

**Supplemental Methods**

**Isolation, Culture, and characterization of rat ADSCs**

Inguinal subcutaneous adipose tissue was acquired under sterile conditions from Sprague-Dawley rats (60-80g) and digested to obtain a stromal vascular fraction (SVF) as previously described with minor modifications . Briefly, adipose was rinsed with sterile phosphate-buffered saline(PBS) , minced and enzymatically dissociated with composite of 0.1% collagnase I (sigma) and 0.05% trypsin (sigma) in serum-free alpha-modified Eagle’s medium (α-MEM, Gibco, USA) for 45-60 min at 37℃ with gentle agitation. Then the enzyme was neutralized with an equal volume of α-MEM/10% fetal bovine serum (FBS, Gibco). The mixture was sequentially filtered through 75μm mesh filter to remove debris and centrifuged at 600g for 5min. the supernatant containing adipocytes and debris was discarded. The pelleted cells was resuspended in α-MEM supplemented with 10% FBS and plated onto 10cm culture plates. The dishes were incubated at 37℃, 5%CO_2_ incubator and the medium was replaced every 2-3 days to remove unattached cells. When cells reached 80-90% confluence within 3-5 days after the initial plating, adherent cells were detached with 0.25% trypsin/0.04% EDTA(v/v 1:1) and seeded at a ratio of 1:3. Cultures were passaged every 3-4 days and used for experimental procedures at passage 3 to 5.

**Flow Cytometry**

To confirm the immunophenotype of adherent cells, surface markers CD34, CD31, CD45, CD90, and CD29 were analyzed by fluorescence-activated cell sorting (FACS) (BD accrri C6). Briefly, cultured adherent cells isolated from adipose tissue were harvested and washed with cell staining buffer (Biolegend). Cell aliquots (1 × 10^6^ cells) were incubated for 30 min at 4 °C with mouse monoclonal antibodies to rat CD34 (Santa Cruz), CD31(clone WM-51), CD45 (Biolgend), CD90 (Biolgend), and CD29 (Biolgend). For unlabeled antibodies, FITC-conjugated secondary antibodies were added. Then cells were incubated for another 30 min at 4°C. Labeled cells were washed in cell staining buffer twice, and then analyzed by FACS. Isotype-identical antibodies served as controls.

**Multi-differentiation assays**

To confirm multipotency of adherent cells, adipogenic and osteogenic differentiation were performed by Alizarin Red staining and Oil Red O staining, respectively. Briefly, for adipogenic differentiation, cells were seeded on the 6-well plate at a density of 1×10^5^ cells/cm^2^. After one day, the medium was replaced by adipogenic differentiation medium containing low-glucose Dulbeco’s modified Eagle’s medium (DMEM) supplemented with 10% FBS, 2 mM L-glutamine, 100 U/mL penicillin, 100 μg/mL streptomycin, 100 μM L-ascorbic acid (Sigma), 1 μM dexamethasone, 0.5 mM 1-methyl-3-isobutylxanthine, and 100 μM indomethacin. Then cells were cultivated for up to 21 days. For osteogenic differentiation, cells were seeded at a density of 1×10^5^/cm^2^. After reaching 100% confluence, cells were incubated in osteogenic differentiation medium (high-glucose DMEM supplemented with 10% FBS, 0.1 μM dexamethasone, 200 μM L-ascorbic acid, and 10 mM *β*-glycerol phosphate (Sigma) for 3 weeks. Osteogenic and adipogenic differentiation medium were changed every 3 days. Verification of adipogenic and Osteogenic differentiation were performed by Oil Red O staining and Alizarin Red staining respectively.

**Construction of lentiviral vectors carrying dual fusion reporter gene**

ADSCs were lentivirally transduced to express both firefly luciferase (fluc) and monomeric red fluorescent protein (mRFP) as described previously . Briefly, Vector DNA containing Flu-mRFP fusion reporter gene, packaging system ps PAX2 and envelop plasmid pMD 2G were kindly provided by Dr. Sam. S. Gambhir (from Stanford University, Radiology Department). Three Vectors were cotransfected into 293T cells using Entranster^TM^–H reagent (Engreen Biosystem Co, Ltd.) according to manufacturer’s instruction. Lentivirus supernatant was concentrated in ultrafiltrate centrifuge tubes at 5000 g. Concentrated virus was titrated on 293T cells and frozen in -70°C for future use.

**Lentiviral labeling of Rat ADSCs**

For transduction, 1 × 10^3^ target cells/cm^2^ at passage 1 were plated in a 6-well cell culture plate. After 60-70% confluence, the cells were incubated with lentiviral vectors (at a multiplicity of infection of 15) in Opti-MEM (Gibco BRL) containing 8 μg/mL polybrene (Sigma) for 24h. After transduction, monomeric red fluorescence protein (mRFP) positive ADSCs were analyzed by FACScan (BD FACSVantage Diva). The 5% highest mRFP expressing cells were selected by FACS and expanded for several passages before usage.

**Body weight measurement before and after Exendin-4 treatment**

Adult male SD rats were maintained in a normal day/night cycle with free access to diet and water. Rats were treated with 1 nM/kg Exendin-4 in PBS daily by intraperitoneal injection starting 3 days before permanent surgical ligation of the left anterior descending artery in the morning daily for 7 days . Body weight was measured before and after Exendin-4 treatment. All procedures were in accordance with the guidelines for care and use of laboratory animals and approved by The Animal Subjects Committee of the General Hospital of Chinese People’s Liberation Amy.

**Myocardial infarction model, cell delivery and Exendin-4 treatment**

Male Sprague-Dawley (SD) rats (250±10g) were purchased from the Experimental Animal Center, Academy of Military Medical Science (Beijing, PRC). All procedures were in accordance with the *Guide for the Care and Use of Laboratory Animals* published by the US National Institutes of Health (NIH Publication, 8th Edition, 2011) and approved by the Institutional Animal Care and Use Committee (IACUC) of the General Hospital of Chinese People’s Liberation Amy. Every effort was made to minimize animal suffering and the number of animals used. Animals were individually housed in cages (accessible to water and food) with a room temperature of 24±2°C (a normal day/night cycle). Rats were randomly divided into the following groups with n=30 each：(1) PBS, (2) Exendin-4 only, (3) ADSCs transplantation, (4) ADSCs transplantation combined with Exendin-4.

Rats were intraperitoneally anesthetized with sodium pentobarbital (30 mg/kg). Limb-lead electrocardiography was performed sequently. The animals were then incubated and ventilated by a volume-regulated respirator during surgery. After a left lateral thoracotomy and pericardectomy, the left coronary artery was identified and gently ligated with a 6.0 prolene suture at approximately 2-3 mm from its origin between the pulmonary artery conus and the left atrium. Successful AMI was confirmed by the typical ST segment elevation in electrocardiography. 5×10^6^ ADSCs in 100μL PBS were injected along peri-infarct zone at three injected foci with a 28-gauge needle. 100 μL PBS alone were injected as control group. Injections were made at an angle to reduce the chance of the injection into the lumen of the LV. Injections were verified by a slight lightening in the color of the myocardium as the solutions entered the infarcted wall. During the surgical procedures, the adequacy of anaesthesia was monitored using absence of the pedal withdrawal reflex, slow constant breathing, and no response to surgical manipulation. Buprenorphine was administered before and after the procedures (0.05 mg/kg, i.p). All the rats received anti-microbial therapy (penicillin, intramuscular twice daily for 3days). As reported previously, treatment with Exendin-4 (Sigma) was intraperitoneally administered daily at a low dose of 1nmol /kg starting 3 days prior to permanent surgical ligation of the left anterior descending artery in the morning for 7days . All rats were treated with cyclosporin A (10 mg/kg/day, ip, Sigma) two days before transplantation and daily until the end of the study.

**Cardiac function assessment with echocardiogram**

Echocardiograms were performed at day 28 post-infarction. Rats were anaesthetized using 1.5–2.0% isofluorane for function measurement with echocardiogram (14.0 MHz, Sequoia 512; Acuson, Germany). LV parameters were obtained from M-mode interrogation in a parasternal long-axis view. Left-ventricular end-diastolic diameter (LVEDD) and left-ventricular end-systolic diameter (LVESD) were measured. LV fractional shortening (FS) and LV ejection fraction (EF) were calculated as follows: FS(%)= [(LVEDD-LVESD)/LVEDD]×100; EF(%)=[(EDD^3^-ESD^3^)/EDD^3^]×100. All measurements were averaged from at least three separate cardiac cycles. All procedures and analyses were performed by an experienced and blinded researcher.

**Histological analysis**

Animals were sacrificed at 1 week after transplantation (n=6/Groups) and at the end of the experiments (n=24/Groups). Hearts were excised and rapidly frozen in O.C.T medium for the preparation of frozen sections (4 μm thickness). For determining infarct size and fibrotic area, ten sections were prepared at 10 different transversal levels at the site of tissue necrosis, equally distributed from base to apex. The sections were stained with HE and Masson’s Trichrome and evaluated using computer assisted planimetry. The infarct size was quantified as the percentage of the total endocardial circumference of left ventricle divided by the infarcted endocardial circumference. The degrees of collagen fiber accumulation in the infarcted area were evaluated by measuring the percentage of fibrotic region in the LV area, which was calculated using RS Image Pro, version 4.5 (Media Cybernetics, Inc., Trenton, NJ).

To determine the inflammatory cell infiltration, heart sections were stained with hematoxylin and eosin. Under high magnification, the densities of lymphocytes and macrophages in the infarcted border zones of the heart were determined manually based on morphology of nuclei and cell size. For each heart sample, 50 random high resolution fields (10 sections at different transversal levels, 5 random fields for each section) were chosen and counted in a blinded manner. In H&E staining, lymphocytes showed less cytoplasm with round or oval hyperchromatic nuclei . Macrophages are oval and measure 9-12 μm in diameter, with dark pink cytoplasm. The nucleus of macrophages is oval or kidney-shaped and generally eccentrically placed .

**Assessment of Intracellular reactive oxygen species (ROS)**

Intracellular ROS were measured by dihydroethidium (DHE) staining. Sections were incubated with DHE (1 mM) for 30 min in dark. After incubation, sections were washed with PBS and counterstained with DAPI for 15min. ROS generation in the border zone of MI in sections was labeled with red fluorescence and examined under fluorescence microscopes (Olympus). For each heart sample, 50 random high resolution fields (10 sections at different transversal levels, 5 random fields for each section) were chosen and counted in a blinded manner. The intensity of DHE staining was quantified using IPWIN60 software (Media Cybernetics, Inc.) expressed as the ratio of DAPI staining.

**TUNEL staining**

Terminal deoxynucleotidyl transferase dUTP nick end labeling (TUNEL) staining was performed on myocardial frozen sections according to the manufacturer’s instructions (MEBSTAIN Apoptosis kit II; Takara). Digital images were acquired at high magnification by using a fluorescent microscopy (Olympus) and the infarct area was manually traced on the blue channel. For each heart sample, 50 random high resolution fields (10 sections at different transversal levels, 5 random fields for each section) were chosen and counted in a blinded manner.

**Immunofluorescence staining**

Vascular density was determined in the section stained with anti-vWF antibody (1:200, Sigma), FITC-conjugated IgG (Sigma, 1:100) were incubated for 1 h at room temperature before observing under an Olympus fluorescent microscopy. The number of capillaries was calculated in five randomly high magnification fields. Microvessels in each section were quantified using the following criteria: a) positive for vessel smooth muscle labeling within peri-infarction region; b) having a visible lumen; and c) having a diameter between 10 and 100 μm. The density of arteriole was expressed as the quantity of arteriole per mm^2^.

The differentiation of ADSCs in the infarcted heart was identified by immunofluorescent staining. Briefly, Animals were euthanized and hearts were explanted and cut into 2 transverseslices through infarct. Then the hearts were frozen in O.C.T. for frozen sections (4 μm) preparation. Ten sections were prepared at 10 different transversal levels at the site of tissue necrosis, encompassing the entire infarcted area. Then cTnT and α-SMA immunohistochemistry was performed, respectively. Immunofluorescent staining was performed on 4 μm sections using following primary antibodies: anti-cardiac troponin T 1:200 (abcam), anti-connexin43 1:500 (cx43, abcam) and anti-α-SMA 1:200 (sigma). Cryosections were fixed with acetone for 30 min and endogenous peroxide activity was quenched with 3% H_2_O_2_. After blocking with 2% normal goat serum, sections were incubated with the primary antibodies at 4°C overnight. Then, FITC-conjugated IgG were incubated for 2 h at room temperature before observing under laser confocal microscope (FV1000, Olympus). Ten high-magnification fields of each section were chosen randomly. The number of the mRPF-positive cardiomyocytes (cTnT+/mRFP+) was calculated. mRPF-positive cardiomyocytes were defined by cells having clear striation staining pattern of cardiac troponin-T. The rate of mRFP-positive cardiomyocytes was defined as the number of the cTnT+ mRFP cells divided by the number of mRFP cells. The number of the mRPF-positive vascular smooth muscle cells (SMA+/mRFP+) was calculated, too. The rate of mRFP-positive vascular smooth muscle cells was defined as the number of the SMA+ mRFP cells divided by the number of mRFP cells.

**Quantitative Real-Time Polymerase Chain Reaction (PCR)**

ADSCs that were treated with or without Exendin-4 for 24h were collected. Total RNA was isolated from cells using TRIzol Reagent (Ambion, Carlsbad CA, USA). First-strand cDNA was synthesized using Thermo First cDNA Synthesis Kit (Germany) according to the standard procedures. The qPCRs were performed in triplicate with the FastStart Universal SYBR Green Master (ROX; Roche, Mannheim, Germany) and run on the StepOnePLUS system (Applied Biosystems, USA). The results were obtained from three independent experiments, whereby a no-template control was included. All primers were designed by the Primer 5 software (Premier Biosoft International, Palo Alto, Canada), which were listed as the following.

| **Gene** | **Primer sequence** | **Product (bp)** |
| --- | --- | --- |
| β-actin | F：GCTACAGCTTCACCACCACA  R：GCCATCTCTTGCTCGAAGTC | 84 |
| bFGF | F：CTGGCTATGAAGGAAGATGG  R：CGTGACCGGTAAGTGTTGTA | 107 |
| HGF | F：GAGAAATGCAGTCAGCACCA  R：TGAGTGGGCCACCATAATCT | 115 |
| IGF-1 | F：GGCATTGTGGATGAGTGTTG  R：GTCTTGGGCATGTCAGTGTG | 128 |
| VEGF | F：AATGATGAAGCCCTGGAGTG  R：ATGCTGCAGGAAGCTCATCT | 114 |

**Western blotting analysis**

The samples of infarcted myocardium (6 rats per group, chosen randomly) and prepared ADSCs were lysed in Laemmli Sample Buffer (Bio-Rad) and further homogenized with a rotorstator homogenizer. Proteins were isolated and concentrations were determined using the BCA^TM^ Protein Assay Kit (Thermo Scientific). 80-120 μg proteins were loaded on a 12-15% sodium dodecyl sulfate-polyacrylamide gel. After electrophoresis, proteins were transferred to a PVDF Western Blotting membrane (Roche). Membrane were blocked with 5% nonfat dried milk (in TBST) for 2h at room temperature and then incubated overnight at 4°C with Primary antibody against TNF-α (R&D, 1:200), Akt1/2/3 (santa cruze. 1:1000), phospho-Akt (santa cruze, 1:1000), ERK (Cell signaling, 1:500), phosphor-ERK (Cell signaling, 1:500), STAT3 (Cell signaling, 1:500), phosphor- STAT3 (Cell signaling, 1:500), Bcl-2 (Santa Cruze, 1:500), Bax (Santa Cruze, 1:500), GAPDH (Cell Signaling Technology, 1:1000), β-actin (Cell Signaling Technology, 1:1000). The membrane was subsequently washed with TBST (5 min×3) and incubated with horseradish peroxidase-conjugated secondary antibodies (Cell Signaling Technology) for 1 h at room temperature. After washing with TBST (5 min×3), bands were detected by enhanced chemiluminescence substrate (Applygen). Bands were visualized using an enhanced chemiluminescence system (Tanon 1600, China). Densitometric analysis of Western blots was carried out using Gel Image System software (version
